# Intrahepatic Cholestasis of Pregnancy Levels of Sulfated Progesterone Metabolites Inhibit Farnesoid X Receptor Resulting in a Cholestatic Phenotype

**DOI:** 10.1002/hep.26055

**Published:** 2013-01-08

**Authors:** Shadi Abu-Hayyeh, Georgia Papacleovoulou, Anita Lövgren-Sandblom, Mehreen Tahir, Olayiwola Oduwole, Nurul Akmal Jamaludin, Sabiha Ravat, Vanya Nikolova, Jenny Chambers, Clare Selden, Myrddin Rees, Hanns-Ulrich Marschall, Malcolm G Parker, Catherine Williamson

**Affiliations:** 1 Institute of Reproductive and Developmental Biology, Dept. of Surgery and Cancer, Faculty of Medicine, Imperial College LondonLondon, UK; 2 Department of Clinical Chemistry, Karolinska University Hospital HuddingeStockholm, Sweden; 3 UCL Institute of Liver and Digestive Health, Royal Free Hospital Campus University College Medical SchoolLondon, UK; 4 North Hampshire Hospital National Health Service TrustBasingstoke, Hampshire, UK; 5 Institute of Medicine, Department of Internal Medicine, Sahlgrenska Academy, University of GothenburgGothenburg, Sweden

## Abstract

Intrahepatic cholestasis of pregnancy (ICP) is the most prevalent pregnancy-specific liver disease and is associated with an increased risk of adverse fetal outcomes, including preterm labor and intrauterine death. The endocrine signals that cause cholestasis are not known but 3α-sulfated progesterone metabolites have been shown to be elevated in ICP, leading us to study the impact of sulfated progesterone metabolites on farnesoid X receptor (FXR)-mediated bile acid homeostasis pathways. Here we report that the 3β-sulfated progesterone metabolite epiallopregnanolone sulfate is supraphysiologically raised in the serum of ICP patients. Mice challenged with cholic acid developed hypercholanemia and a hepatic gene expression profile indicative of FXR activation. However, coadministration of epiallopregnanolone sulfate with cholic acid exacerbated the hypercholanemia and resulted in aberrant gene expression profiles for hepatic bile acid-responsive genes consistent with cholestasis. We demonstrate that levels of epiallopregnanolone sulfate found in ICP can function as a partial agonist for FXR, resulting in the aberrant expression of bile acid homeostasis genes in hepatoma cell lines and primary human hepatocytes. Furthermore, epiallopregnanolone sulfate inhibition of FXR results in reduced FXR-mediated bile acid efflux and secreted FGF19. Using cofactor recruitment assays, we show that epiallopregnanolone sulfate competitively inhibits bile acid-mediated recruitment of cofactor motifs to the FXR-ligand binding domain. *Conclusion*: Our results reveal a novel molecular interaction between ICP-associated levels of the 3β-sulfated progesterone metabolite epiallopregnanolone sulfate and FXR that couples the endocrine component of pregnancy in ICP to abnormal bile acid homeostasis. (Hepatology 2013;)

Intrahepatic cholestasis of pregnancy (ICP) is the commonest pregnancy-specific liver disease. It typically affects 0.5%-1.5% of pregnant women in Europe and is 2-3 times more prevalent in women of Chilean and Asian origin.^1^ ICP presents in the second or third trimester of pregnancy with maternal pruritus, raised liver transaminases, and serum bile acids.^1^ It may be complicated by fetal hypoxia, spontaneous preterm labor, and intrauterine death.^2^,^3^ Genetic variation has been reported in bile acid homeostasis-related genes, including the hepatic bile acid receptor, farnesoid X receptor (FXR; *NR1H4*)^4^ and its target genes, the bile salt export pump (BSEP; *ABCB11*),^5^,^6^ MDR3 (*ABCB4*),^7^-^10^ and MRP2 (*ABCC2*).^11^ However, women with ICP are usually asymptomatic when they are not pregnant and the disease phenotype is unmasked by pregnancy in the majority of cases. The symptoms and biochemical features of ICP usually resolve after delivery of the fetus, although ∼ 15% of affected women also develop cholestasis when given exogenous estrogens^12^ or the combined oral contraceptive pill.^13^ Thus, it is likely that reproductive hormones play a role in the etiology of ICP.

3α-Sulfated progesterone metabolites are raised in the serum and urine of women with ICP compared to those with uncomplicated pregnancy.^14^-^17^ Maximal serum concentrations for 3α-monosulfated and -disulfated progesterone metabolites that are elevated in ICP have been shown to reach 4.5 μM and 12 μM, respectively.^15^ Furthermore, these metabolites have been shown to be raised before the onset of the disease.^18^ It has been demonstrated that sulfated progesterone metabolites impair hepatic bile acid transport. We have recently shown that two monosulfated progesterone metabolites, allopregnanolone sulfate (PM4S) and epiallopregnanolone sulfate (PM5S), reduce Na^+^-dependent and Na^+^-independent influx of the primary bile acid taurocholate into primary human hepatocytes.^19^ They were shown to competitively inhibit taurocholate uptake by the principal sinusoidal bile acid transporter, Na^+^-taurocholate cotransporting polypeptide (NTCP), in NTCP-transfected *Xenopus laevis* oocytes^19^ and to reduce the efflux of bile acids from *X. laevis* oocytes expressing BSEP.^20^ Although it has been demonstrated that sulfated progesterone metabolites directly impair biliary transport of bile acids, it has not been established whether they influence hepatic pathways of bile acid homeostasis.

The nuclear receptor FXR plays a central role in hepatic bile acid homeostasis. In the presence of raised hepatocyte bile acid levels, FXR heterodimerizes with the retinoid X receptor (RXR) and regulates bile flow by inducing the expression of the canalicular transporters that mediate efflux of bile acids (*BSEP*), organic anions (*MRP2),* and phosphatidylcholine (*MDR3*) into bile.^21^,^22^ Another important FXR target gene is the short heterodimer partner (*SHP*), which represses *NTCP*^23^ and enzymes in the bile acid synthetic pathway (*CYP7A1, CYP7B1*).^24^ Thus, FXR prevents the accumulation of bile acids in hepatocytes by regulating their uptake, synthesis, and export.

We have shown that FXR function is impaired in murine pregnancy,^25^ and therefore we hypothesize that the levels of sulfated progesterone metabolites found in ICP abrogate FXR function despite the presence of raised serum and hepatocyte bile acids. Here we demonstrate that levels of the 3β-sulfated progesterone metabolite, epiallopregnanolone sulfate (PM5S), are supraphysiologically raised in ICP. Moreover, we show that PM5S can exacerbate cholic acid-induced hypercholanemia in the mouse and inhibit the hepatic induction of FXR target genes, thus reducing the expression and function of BSEP, and providing an explanation for the development of maternal cholestasis in ICP.

## Materials and Methods

### Human Serum Samples

This study conformed to the 1975 Declaration of Helsinki guidelines and permission was obtained from the Ethics Committee of the Hammersmith Hospitals NHS Trust, London (97/5197 and 08/H0707/21). Blood was collected from 15 women with ICP and 12 controls with uncomplicated pregnancy (between 33-41 weeks) (see Supporting Table 1 for patient details). Diagnostic criteria and sample preparation were as described.^19^,^26^

#### Animals and Treatments

Studies were conducted in accordance with the UK Animals (Scientific Procedures) Act of 1986. The 7 to 8-week-old C57BL6 female mice were administered by oral gavage either 200 μL of 20% cyclodextrin (vehicle), 50 mg/kg of cholic acid, or 50 mg/kg of cholic acid and 500 mg/kg of PM5S twice daily for 2 days: day 1 at 5:30 pm and 10:30 pm, day 2 at 8:30 AM and 1:30 pm. On the second day, tissues were harvested at 4 pm after an 8-hour fast.

Methods for further biochemical, molecular, and *in vitro* cell-culture experiments are described in the Supporting Information.

## Results

### Levels of the 3β-Sulfated Progesterone Metabolite Epiallopregnanolone Sulfate Are Raised in ICP

**Fig. 1:**
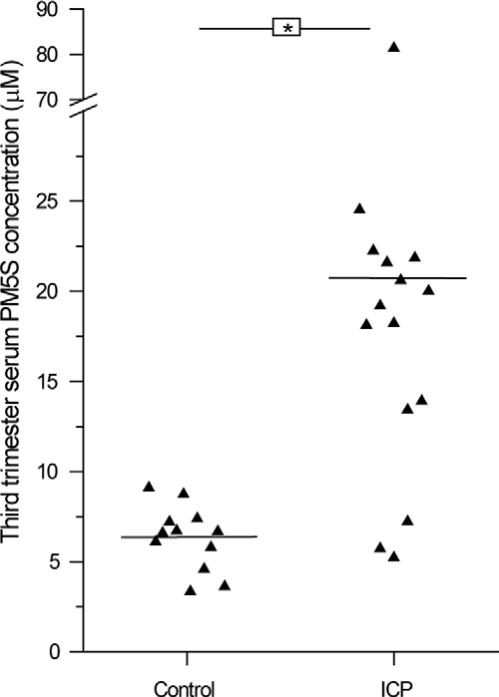
Levels of epiallopregnanolone-sulfate (PM5S) are supraphysiologically raised in ICP. Serum concentrations of PM5S in women with ICP and uncomplicated pregnancies at 33-38 weeks of gestation. Black line represents mean serum concentrations of PM5S. Measurements were carried out by UPLC/MSMS on a minimum of n = 12 samples. *P < 0.05 for control pregnant versus ICP serum samples as determined by Student's *t* test.

We have previously shown that the levels of the sulfated progesterone metabolite epiallopregnanolone sulfate (PM5S) are increased in normal pregnancy relative to nonpregnant women.^19^ To investigate whether the levels of PM5S are further raised in ICP patients, UPLC/MSMS was used to assay PM5S serum concentrations in pregnant women with ICP or uncomplicated pregnancy (33-41 weeks). Serum from control and ICP cases had mean concentrations of PM5S of 6.3 μM and 21 μM, respectively (Fig. 1), corresponding to a significant 330% increase in PM5S levels in ICP. This result demonstrates for the first time that a 3β-sulfated progesterone metabolite is supraphysiologically raised in ICP at concentrations greater than those reported for the 3α-sulfated progesterone metabolites.^15^,^18^

#### Raised Serum Bile Acids in PM5S-Challenged Mice

**Fig. 2:**
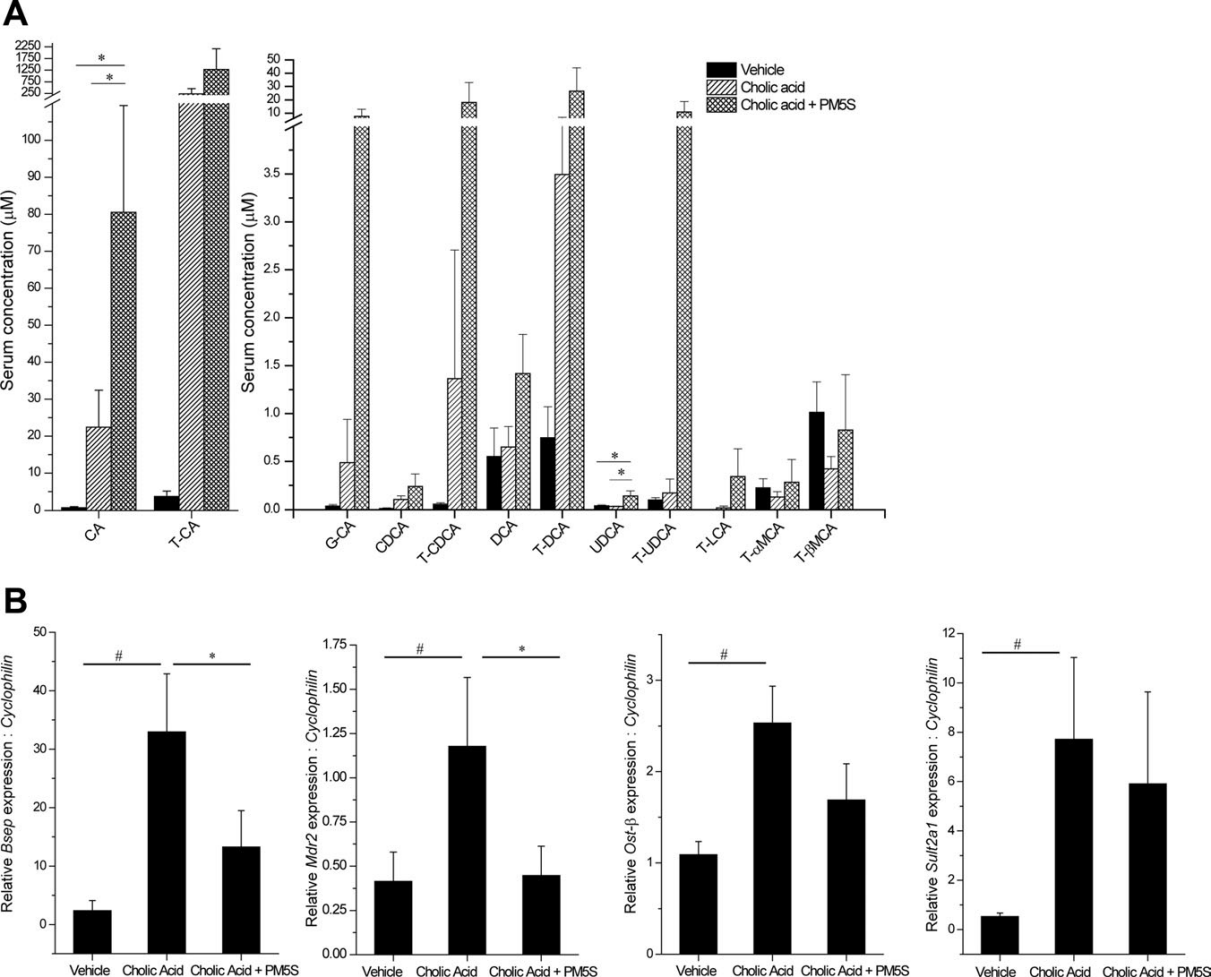
PM5S exacerbates hypercholanemia in the mouse. (A) UPLC/MSMS-derived serum bile acid concentrations represented in two graphs according to the range of their concentrations and (B) hepatic gene expression levels of *Bsep, Mdr2, Ost-β*, and *Sult2a1* in mice gavaged with vehicle, cholic acid, or cholic acid and PM5S at four timepoints over 2 days. *P < 0.05 for vehicle or cholic acid-gavaged group versus cholic acid and PM5S co-gavaged group. ^#^*P* < 0.05 for vehicle versus cholic acid-gavaged group as determined by one-way analysis of variance (ANOVA). Values represent mean ± standard error of the mean (SEM) of n = 6. CA, cholic acid; T-CA, taurocholic acid; G-CA, glycocholic acid; CDCA, chenodeoxycholic acid; T-CDCA, taurochenodeoxycholic acid; DCA, deoxycholic acid; UDCA, ursodeoxycholic acid; T-UDCA, tauroursodeoxycholic acid; T-LCA, taurolithocholic acid; T-αMCA, tauro-α muricholic acid; T-βMCA, tauro-β muricholic acid.

To investigate whether PM5S can interfere with bile acid homeostasis, we studied the impact of PM5S on the ability of a mouse to metabolize cholic acid by comparing bile acid and gene expression levels in mice orally gavaged with either vehicle, cholic acid (CA), or CA and PM5S (CA+PM5S). Mice coadministered CA+PM5S had significantly raised serum PM5S (Table 1) and CA levels, and a trend for conjugated bile acids to be raised when compared to the vehicle or CA groups (Fig. 2A). Hepatic gene expression levels of *Bsep, Mdr2, Ost-β*, and *Sult2a1* were significantly induced in the CA group (Fig. 2B). In contrast, induction of *Bsep* and *Mdr2* expression was significantly abrogated and there was a trend for *Ost-β* and *Sult2a1* expression to be reduced in the CA+PM5S group when compared to the CA group. There were no differences in *Shp* and *Ntcp* expression levels (Supporting Fig. 1). These data establish that PM5S can interfere with bile acid metabolism resulting in hypercholanemia and impaired induction of key hepatic bile acid-responsive genes consistent with cholestasis.

**Table 1:** PM5S Concentrations Observed in Mice Gavaged with Vehicle, Cholic Acid or Cholic Acid and PM5S

	Mean Concentration (μM) ± SEM		
	
Serum Compound	Vehicle	Cholic Acid	Cholic Acid + PM5S
PM5S	4.9 ± 3.9	10.7 ± 9.6	3208.6 ± 1847.1*

* *P* < 0.05 for cholic acid versus cholic acid + PM5S, as determined by one-way ANOVA. Values represent mean ± SEM of n ≥ 6.

#### PM5S Inhibits FXR-mediated BSEP Expression and Function

**Fig. 3:**
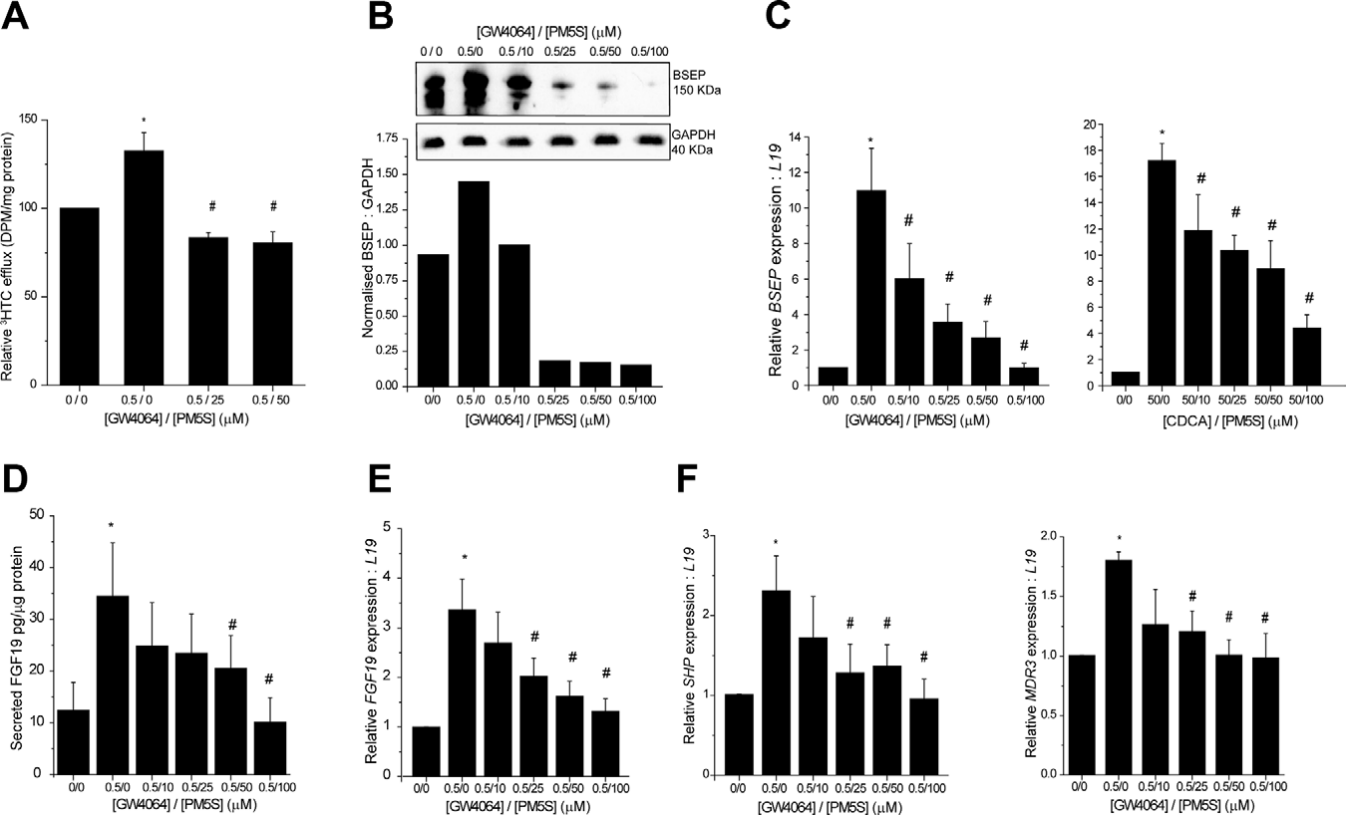
PM5S inhibits function and expression of FXR target genes. (A) Huh7 cells were treated overnight with vehicle or 0.5 μM GW4064 ± 25-50 μM PM5S, following which cells were washed for 1.5 hours and allowed to efflux^3^HTC for 5 minutes. (B) Representative western blot and graphical representation of normalized BSEP to GAPDH levels in Huh7 cells treated with vehicle or 0.5 μM GW4064 ± 0-100 μM PM5S for 24 hours. (C) Huh7 cells were treated with vehicle or 0.5 μM GW4064 / 50 μM CDCA ±0-100 μM PM5S for 24 hours, after which cells were analyzed with qPCR for relative *BSEP* gene expression. (D) GW4064-mediated secretion of FGF19 is inhibited by PM5S. Secreted FGF19 was assayed in cell culture media taken from Huh7 cells incubated with 0.5 μM GW4064 ±0-100 μM PM5S for 24 hours. (E) GW4064-mediated *FGF19* gene expression is inhibited by PM5S. Experiments were performed as in (C). (F) GW4064-mediated *SHP* and *MDR3* gene expression is inhibited by PM5S. Experiments were performed as in (C). *P < 0.05 for 0.5 μM GW4064/50 μM CDCA versus vehicle control. ^#^*P* < 0.05 for 0.5 μM GW4064/50 μM CDCA versus cotreatment group as determined by one-way ANOVA. Values represent mean ± SEM of n = 3.

We addressed the hypothesis that the increase in the levels of serum PM5S in ICP may abrogate FXR function by testing its effects on FXR-mediated bile acid transport. Huh7 cells were pretreated with the FXR agonist GW4064 ±PM5S for 24 hours, following which^3^H-taurocholic acid (3HTC) uptake and efflux were assessed. Intracellular^3^HTC levels did not vary significantly between all the treatment groups studied prior to the efflux assay (data not shown). However,^3^HTC efflux was significantly increased in GW4064-treated cells relative to the vehicle treated group, an increase that was abolished when cotreated with 25 or 50 μM PM5S (Fig. 3A). These data demonstrate that supraphysiological levels of PM5S, at concentrations similar to those observed in ICP, are capable of perturbing FXR-mediated bile acid efflux.

Bile acid efflux has been shown to be primarily mediated by BSEP.^27^ BSEP expression was investigated in Huh7 cells that were treated with GW4064 ±increasing doses of PM5S. GW4064 treatment resulted in a 1.5-fold increase in BSEP protein levels relative to vehicle-treated control cells. The addition of 10 μM PM5S inhibited the GW4064-mediated BSEP protein expression. Furthermore, GW4064 ±25-100 μM PM5S treatment completely abolished BSEP protein expression (Fig. 3B). Similarly GW4064-mediated *BSEP* gene expression was completely abolished by the addition of increasing PM5S doses, whereas PM5S significantly reduced CDCA-mediated *BSEP* expression at all concentrations tested, but without completely blunting the CDCA-induced component of expression (Fig. 3C). Importantly, the expression of BSEP was markedly reduced at both 10 and 25 μM PM5S, the levels observed in the majority of women with ICP, and therefore can account for, at least in part, the inhibition of GW4064-mediated bile acid efflux.

We investigated the expression of FGF19 in the Huh7 cells, as this has been shown to be an FXR target in human hepatocytes.^28^ GW4064 treatment resulted in a significant 2.8-fold increase in secreted FGF19. Cotreatment with 10 μM and 25 μM PM5S resulted in a trend towards a reduction in the levels of secreted FGF19, reaching significance with the addition of 50 μM and 100 μM PM5S (Fig. 3D). Similarly, the addition of 25-100 μM PM5S significantly reduced GW4064-mediated *FGF19* gene expression in a dose-dependent manner (Fig. 3E). The expression of the FXR target genes, *SHP* and *MDR3* was regulated in the same manner by PM5S, such that the GW4064-mediated *SHP* and *MDR3* gene expression was significantly abrogated by PM5S in a dose-dependent manner (Fig. 3F). These data were confirmed in primary human hepatocytes with the exception of *SHP* (Supporting Fig. 2). A comparison of the cycle threshold values of quantitative polymerase chain reaction (qPCR) with those obtained from Huh7 cells reveal similar expression levels (Supporting Table 2).

We investigated whether PM5S exhibits any partial agonist activity by analyzing *BSEP* and *SHP* expression in primary human hepatocytes treated with PM5S or CDCA. *BSEP* expression was induced 7.5-fold by the FXR ligand CDCA, but it was also modestly increased by PM5S (Supporting Fig. 3A). PM5S was capable of inducing *SHP* expression, albeit to a lesser extent than CDCA (Supporting Fig. 3B). Thus, we conclude that PM5S is an antagonist of FXR with partial agonist activity.

#### Sulfated Progesterone Metabolites Are Competitive Inhibitors of Ligand-Dependent FXR Activation

**Fig. 4:**
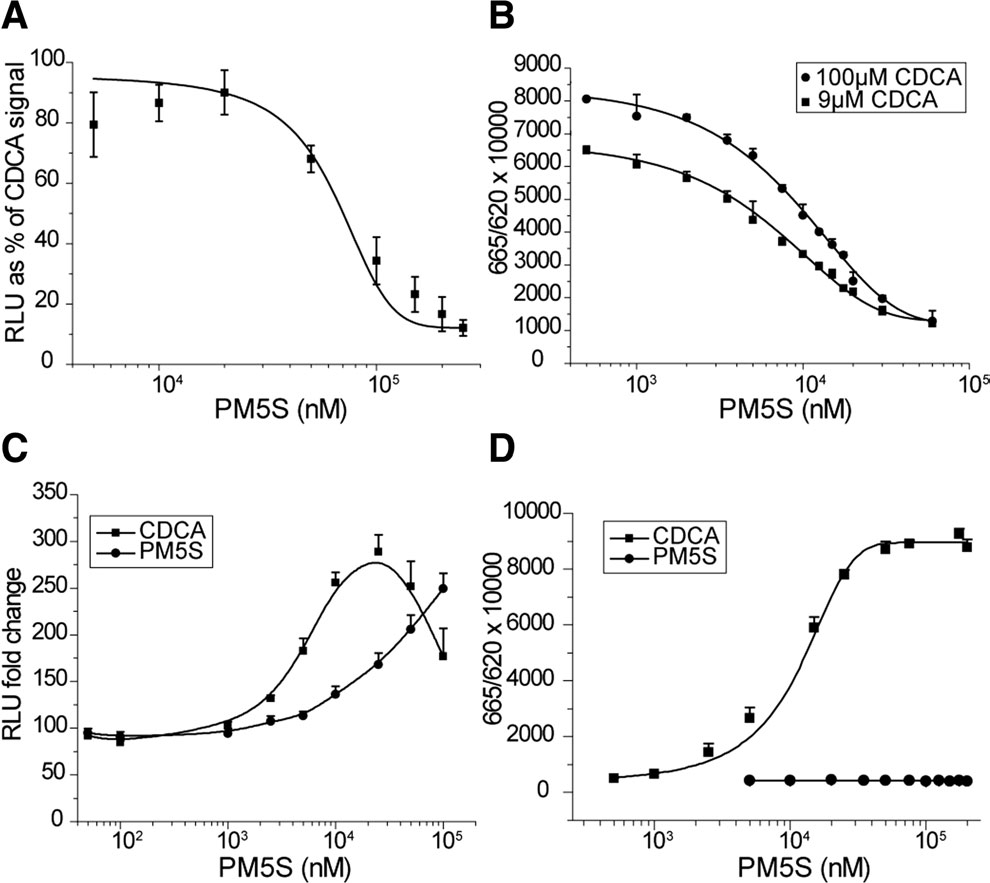
PM5S reduces ligand-mediated FXR activity. (A) Huh7 cells were transfected with the human expression constructs RXR, FXRα2 / empty vector, FXR-luciferase reporter and renilla construct for 24 hours. Transfected cells were cotreated with 100 μM CDCA and increasing doses of PM5S. RLU, relative light units. n = 3 ± SEM. (B) PM5S inhibits ligand activated FXR recruitment of SRC-1. GST-tagged FXR-LBD and biotinylated LxxLL SRC-1 peptide incubated in the presence of 9 or 100 μM CDCA and 0-600 μM PM5S and HTRF measured after 1 hour incubation at room temperature with orbital shaking. n = 3 ± standard deviation (SD). (C) PM5S exhibits dose-dependent mild FXR agonistic characteristics. Transfection experiments were performed as in (A). Huh7 cells were treated with 0-100 μM PM5S. RLU, relative light units. n = 3 ± SEM. (D) PM5S is unable to recruit the LxxLL motif to the FXR-LBD. Experiments were performed as in (B). HTRF was measured following a 1-hour incubation of HTRF mixture and increasing CDCA/PM5S doses. n = 3 ± SD.

To investigate the molecular mechanism by which PM5S inhibits FXR target gene expression, we used reporter assays to examine FXR activity in Huh7 cells in the presence of the FXR agonist CDCA ±PM5S. CDCA mediated a 3.5-fold increase in FXR transactivity (data not shown), which was abrogated by PM5S concentrations of ≥5 μM in a dose-dependent manner (Fig. 4A). This was confirmed using a BSEP-promoter-luciferase construct (Supporting Fig. 4). This result proved that FXR activity is sensitive to the actions of PM5S at concentrations found in ICP.

Given that ligand binding to the ligand-binding domain (LBD) of many nuclear receptors leads to the recruitment of transcriptional cofactors by means of LxxLL motifs,^29^ we established a cell-free homogeneous time-resolved fluorescence (HTRF) cofactor recruitment assay for the FXR-LBD. In this assay the fluorescence resonance energy transfer resulting from the binding of a fluorescently labeled LxxLL containing peptide derived from SRC-1 to a GST tagged-FXR-LBD was monitored in the presence of CDCA. The SRC-1 peptide was recruited to the FXR-LBD in a dose-dependent manner with an EC_50_ value of 9 μM and maximal normalized HTRF signal at 100 μM (data not shown), as previously reported.^30^ We next investigated the effect of PM5S on the recruitment of SRC-1 peptide achieved in the presence of CDCA. Based on the EC_50_ and maximal HTRF signal values in the presence of CDCA, we incubated GST-FXR-LBD with SRC-1 peptide in the presence of 9 μM or 100 μM of CDCA together with increasing concentrations of PM5S. We found that PM5S was able to inhibit the CDCA-mediated recruitment of the SRC-1 peptide to the FXR-LBD in a dose-dependent manner (Fig. 4B). Together, these data demonstrate that PM5S can directly antagonize FXR activity by competing with CDCA for the FXR-LBD and, as a consequence, inhibit the recruitment of cofactors possessing the LxxLL motif.

PM5S was able to transactivate FXR in a reporter assay in a dose-dependent manner, but was less efficacious than CDCA (Fig. 4C), generating an EC_50_ of >83 μM, compared to 4.6 μM for CDCA. Surprisingly, in contrast to CDCA, PM5S was unable to potentiate the recruitment of the SRC-1 peptide to the FXR-LBD in an HTRF cofactor peptide recruitment assay (Fig. 4D). These data establish that the progesterone metabolite PM5S functions as a partial agonist of FXR by competitively inhibiting ligand mediated activation. Thus, we conclude that the ability of PM5S to inhibit the recruitment of LxxLL peptides provides a mechanism for the antagonistic activity of PM5S but the molecular basis for the mild partial agonist activity has yet to be established.

#### 3-Carbon β-Sulfated Progesterone Metabolites Are Modulators of FXR

**Fig. 5:**
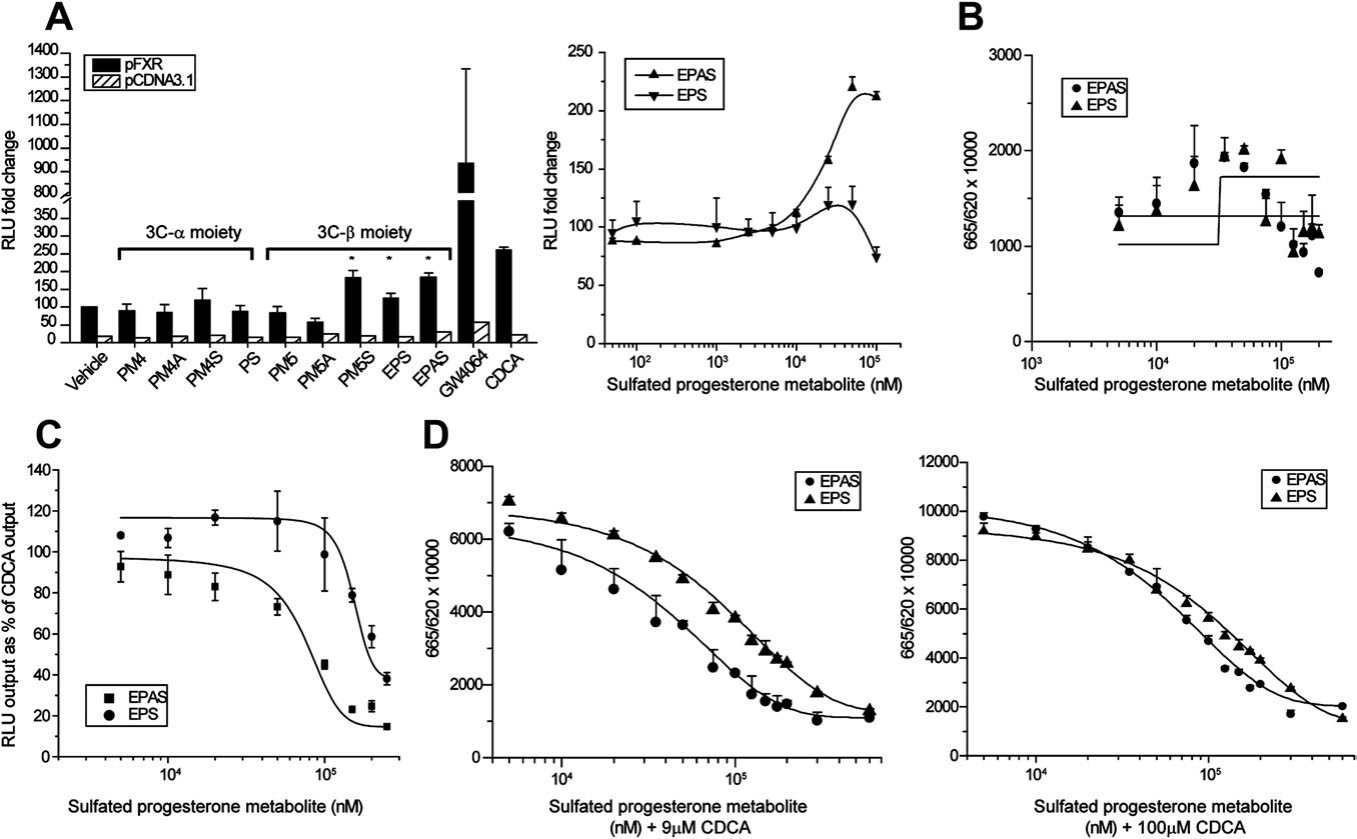
3β-Sulfated progesterone-based compounds modulate FXR activity. (A) Huh7 cells were transfected with the human expression constructs for RXR, FXRα2 / empty vector, FXR-luciferase reporter, and renilla construct for 24 hours. Fifty μM of compound or 1 μM GW4064 was used to treat the transfected cells for 24 hours. *P < 0.05 for treatment group versus vehicle control. RLU, relative light units. n = 3 ± SEM. Dose response curves of 0-100 μM EPAS and EPS, which were identified in the compound screen. RLU, relative light units. n = 3 ± SEM. (B) EPAS and EPS are unable to recruit the LxxLL motif to the FXR-LBD. GST-FXR-LBD and biotinylated LxxLL SRC-1 peptide were incubated in the presence of increasing EPAS, EPS, and CDCA concentrations and HTRF measured after 1-hour incubation at room temperature with orbital shaking. n = 3 ± SD. (C) Huh7 cells were transfected as in (A) and cotreated with 100 μM CDCA and increasing doses of EPAS and EPS. n = 3 ± SEM. (D) EPAS and EPS inhibit ligand-activated FXR recruitment of SRC-1. HTRF experiments were performed as in (B). HTRF reaction mix was incubated in the presence of 9 or 100 μM CDCA and 0-600 μM EPAS/EPS. n = 3 ± SD.

To gain an insight into the structure-function relationship of sulfated progesterone metabolites and FXR, PM5S and a panel of progesterone-based compounds (Supporting Fig. 5) were assayed for their ability to activate FXR in a reporter assay. PM5S, epipregnanolone sulfate (EPS), and epiallo-pregnanediol 3-sodium sulfate (EPAS) were able to significantly transactivate FXR by 182%, 125%, and 183%, respectively, relative to vehicle-treated control (Fig. 5A). These data indicate that progesterone metabolites that have a sulfate group at the 3-carbon of the steroid ring in the β-position are able to function as FXR modulators. EPAS and EPS were able to transactivate FXR in a dose-dependent manner, generating EC_50_ values of 22.6 μM and 15.1 μM, respectively (Fig. 5A), which are lower than the value observed for PM5S. In an HTRF cofactor peptide recruitment assay, EPAS and EPS were unable to induce the recruitment of the SRC-1 peptide to the FXR-LBD (Fig. 5B), consistent with the results obtained for PM5S.

The antagonistic activity of EPAS and EPS was also examined in the FXR reporter assay and HTRF cofactor recruitment assay. In the presence of CDCA, both EPAS and EPS inhibited the activity of FXR (Fig. 5C) and the recruitment of the LxxLL SRC-1 peptide (Fig. 5D) in a dose-dependent manner, demonstrating antagonistic properties. These data demonstrate that FXR-LBD-mediated activity is sensitive to progesterone metabolites sulfated at the 3-carbon in the β-position and that these progesterone-based compounds can function as antagonists with weak agonist activity.

#### Modulation of Endogenous FXR Bile Acid Homeostasis Targets by PM5S

**Fig. 6:**
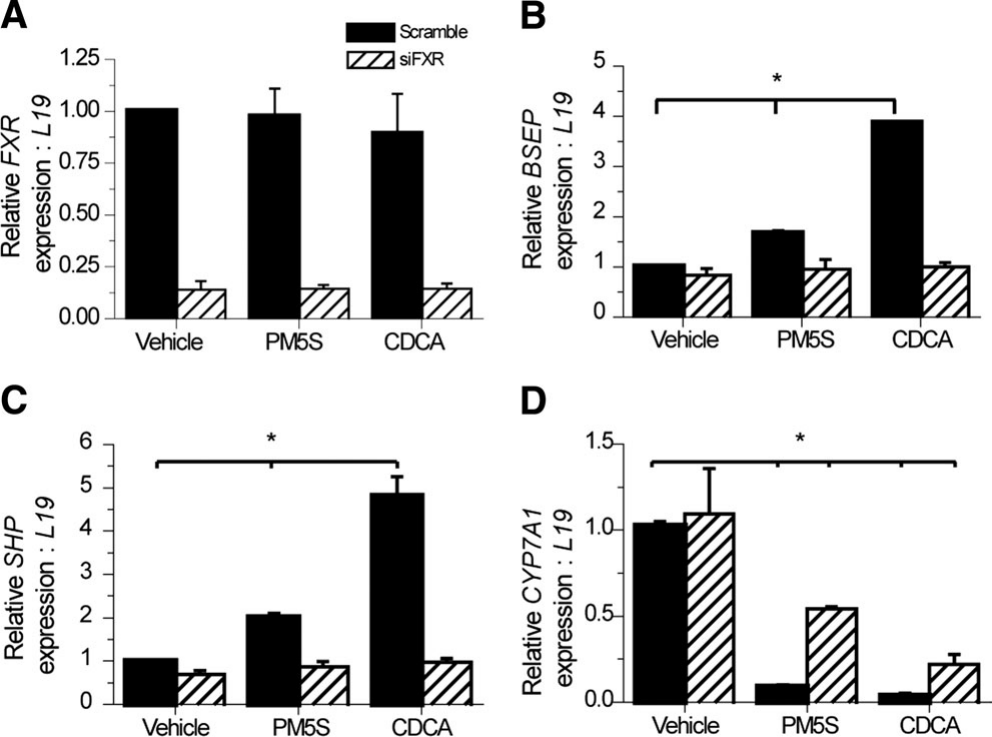
Depletion of FXR in primary human hepatocytes blunts the response to PM5S and CDCA. Primary human hepatocytes were transfected with siRNA against FXR (hatched bar) or scrambled siRNA (black bar) for 6 hours, following which cells were allowed to recover overnight and then treated with 50 μM PM5S or 50 μM CDCA for 24 hours. Relative messenger RNA (mRNA) expression levels are shown for the genes (A) *FXR*, (B) *BSEP*, (C) *SHP*, and (D) *CYP7A1*. *P < 0.05 for treatment group versus vehicle siScrambled vehicle control as determined by one-way ANOVA. Values represent mean ± SEM of n = 3.

To confirm that the effects of the progesterone metabolites are mediated by FXR itself, we depleted primary human hepatocytes of FXR using small interfering RNA (siRNA). We were unable to test the FXR-dependent antagonistic effects of PM5S, as the depletion of FXR resulted in the complete blunting of the CDCA-mediated *BSEP* and *SHP* induction. We therefore examined the effects of 50 μM PM5S or CDCA on FXR target gene expression. FXR RNA levels were reduced by 80% compared with a nontargeting scrambled siRNA in all treatment groups (Fig. 6A). As expected, in the scrambled siRNA group, PM5S and CDCA significantly induced *BSEP* gene expression by 160% and 390%, respectively, but this induction was blunted in cells depleted of FXR to levels similar to those observed in the vehicle-treated group (Fig. 6B). Similarly, *SHP* gene expression was significantly induced in the siScrambled PM5S and CDCA-treated groups by 200% and 481% compared to the vehicle-treated control group but significantly blunted after FXR depletion (Fig. 6C). PM5S and CDCA treatment resulted in the repression of the indirect FXR target *CYP7A1* expression by >90% in the siScramble group but was less marked following FXR depletion (Fig. 6D). Thus, PM5S influences FXR signaling directly and thereby modulates the expression of genes required for bile acid homeostasis.

## Discussion

Our results conclusively show that ICP is associated with supraphysiological levels of the 3β-sulfated progesterone metabolite epiallopregnanolone sulfate (PM5S) and demonstrate potential mechanisms to explain gestational hypercholanemia and cholestasis. The data also reveal a unique interaction between progesterone metabolites that are sulfated at the 3-carbon in the β-position, including PM5S at levels observed in ICP, and the hepatic bile acid nuclear receptor FXR, resulting in perturbed downstream FXR target gene expression and function. FXR plays a major role in the control of serum bile acid levels. It is activated by bile acids in an LBD-dependent manner^31^-^33^ and induces the expression of target genes, including *BSEP, FGF19* and *SHP*, by binding to FXR-response elements.^21^,^24^,^28^ Reduced FXR function has been shown to be associated with high serum bile acid levels in FXR null mice,^34^ in pregnant wildtype mice,^25^ and in a subset of ICP patients with a mutation in the coding region of the FXR DNA-binding domain.^4^

We hypothesized that the cholestatic phenotype that presents as a defining feature of ICP may be due to impaired FXR function, resulting from the abnormally high levels of progesterone metabolites in ICP. Therefore, we investigated the impact of PM5S on FXR activity in the context of ICP.

It has been previously shown that 3α-sulfated progesterone metabolites are raised in ICP.^15^-^18^ In this report, we show for the first time that PM5S, a 3β-monosulfated progesterone metabolite, is significantly raised in women with ICP at concentrations greater than those of each individual sulfated progesterone metabolite species previously documented to be elevated.^15^,^18^ We induced hypercholanemia in mice, as normal murine pregnancy is hypercholanemic,^25^ and found that administering PM5S further increased serum bile acids. In parallel, cholic acid induction of bile acid-responsive genes was perturbed by PM5S, which is likely to explain the observed exacerbation of hypercholanemia and cholestasis. Furthermore, we demonstrate that PM5S found at levels associated with ICP can inhibit GW4064-mediated bile acid efflux by abrogating FXR induction of BSEP expression. PM5S inhibition of BSEP induction and function is likely to play a role in the pathogenesis of ICP, as BSEP activity is the rate-limiting step for hepatic bile acid clearance.^35^ Additionally, BSEP mutations have been reported in ICP cases and in individuals with the nongestational cholestatic conditions, progressive familial intrahepatic cholestasis and benign recurrent intrahepatic cholestasis type 2.^5^,^6^,^36^ These findings can also explain why cholestatic symptom severity in ICP is at its greatest in the third trimester, when progesterone levels are also at their highest.^1^ Mutations in *MDR3* have been shown to be associated with ICP.^9^,^10^ Accordingly, we show that cholic acid/FXR-mediated *Mdr2/MDR3* expression is impaired by PM5S. Additionally, *FGF19* and *SHP* expression is perturbed by PM5S concentrations observed in our ICP patient cohort. The decrease in *FGF19* expression together with the observed PM5S-mediated inhibition of FXR-induced *SHP* expression may result in elevated levels of CYP7A1, the rate-limiting enzyme in the bile acid biosynthetic pathway, as both SHP and FGF19 repress its expression.^24^,^28^ To date, there have been few reports of an endogenous non–bile acid ligand capable of inhibiting FXR activity. Several polyunsaturated fatty acid species have been shown to differentially modulate the downstream expression of FXR targets, enhancing and inhibiting agonist-induced BSEP and kininogen expression, respectively.^37^ We have demonstrated that bile acid-mediated FXR activity is reduced by sulfated progesterone metabolites, at levels consistent with those found in ICP, and that these compete with bile acids for the FXR-LBD, resulting in the inhibition of cofactor recruitment to the FXR-LBD. There are several coactivators that contain the LxxLL motif,^38^ which mediate the FXR signal to enhance transcription. Therefore, impaired recruitment of essential cofactors will in turn reduce FXR target gene expression. This mechanism could in part explain how the hormonal milieu during pregnancy unmasks the cholestatic phenotype of ICP in susceptible women with genetic variation in bile acid homeostasis genes such as *ABCB11, ABCB4,* and *FXR*.^4^-^10^

In common with many well-documented competitive inhibitors,^30^,^39^ we ascertained that sulfated progesterone metabolites that are able to competitively inhibit bile acid-mediated FXR activation also possess mild agonistic activity. A screen of progesterone-based compounds specifically revealed that those that possess a sulfate group at the 3-carbon in the β-position of the steroid ring are able to transactivate FXR in a dose-dependent manner. This is intriguing, as bile acids that modulate FXR have 3-carbon hydroxyl groups, whereas PM5S, EPAS, and EPS have 3-carbon sulfate groups. Moreover, it has been proposed that the human FXR-ligand binding pocket has evolved to accommodate bile salts that possess the *cis* conformation, i.e., the 5-carbon hydrogen in the β-position,^40^ generating an overall bent conformation. On the other hand, PM5S and EPAS possess a hydrogen at the 5-carbon in the α-position (*trans*), giving the overall steroid structure a planar conformation. The mode of interaction between the sulfated progesterone metabolites and the FXR-LBD may lead to the formation of an AF2 surface that is incapable of interacting with coactivators that rely on LxxLL motifs. Accordingly, sulfated progesterone metabolites identified as activators of FXR were unable to recruit the SRC-1 LxxLL motif to the FXR-LBD. Presumably this altered conformation does not prevent the recruitment of coactivators elsewhere on the receptor or alternatively is capable of binding to novel proteins/coactivators which may not normally be reported to interact with the AF2 domain. Similarly, polyunsaturated fatty acids and catechin, a tea-derived compound, were also shown to activate FXR, while unable to potentiate the recruitment of a peptide containing the LxxLL motif to the FXR-LBD.^37^,^41^ The data presented demonstrate that FXR is sensitive to sulfated progesterone metabolites with an atypical steroid backbone structure and sidechain group and that the interaction between the two is capable of perturbing bile acid homeostasis and thus contribute to the etiology of ICP.

It is not known why sulfated progesterone metabolite levels are found at supraphysiological concentrations in ICP, but it has been suggested that excretion of sulfated progesterone metabolites may be impaired due to aberrant hepatobiliary excretion and or dysregulated phase II metabolism of progesterone.^17^ Ursodeoxycholic acid is commonly used to treat ICP and in the majority of cases it ameliorates symptoms and in parallel reduces serum and urinary levels of sulfated progesterone metabolites and bile acids.^42^,^43^ This suggests that sulfated progesterone metabolites and bile acids share common ursodeoxycholic acid-mediated pathways of metabolism or excretion.

Women with ICP have an increased risk of developing gallstones.^13^ Given that women with ICP are hypercholesterolemic^44^ and that the type of gallstones associated with ICP are cholesterol-rich,^45^ it is conceivable that raised serum levels of sulfated progesterone metabolites enhance the formation of gallstones by disrupting cholesterol and bile acid metabolism pathways modulated by FXR. Consistent with this, it has been shown that FXR activation in the mouse liver ameliorates cholesterol gallstones.^46^

The fetal complications of ICP are more prevalent in pregnancies with higher maternal serum bile acid levels,^3^ and any therapeutic intervention that reduces maternal bile acids is likely to reduce the risk of preterm labor, fetal hypoxia, and intrauterine death in affected pregnancies. In this study, we identify PM5S, a progesterone metabolite, as being supraphysiologically raised in women with ICP. We also show that levels of PM5S found in ICP are capable of reducing the function of the bile acid nuclear receptor FXR and therefore impact the downstream FXR bile acid homeostasis target function. This has important ramifications for the etiology of ICP, as it pairs the endocrine environment of pregnancy to the dysregulation of bile acid homeostasis in susceptible women. This highlights a novel FXR interaction as being a potential therapeutic target for the treatment of cholestasis. By implication, this may also improve the fetal outcome in ICP pregnancies by reducing maternal serum bile acid levels.

## Abbreviations

BSEP: bile salt export pump
CA: cholic acid
CDCA: chenodeoxycholic acid
FGF19: fibroblast growth factor 19
FXR: farnesoid X receptor
ICP: intrahepatic cholestasis of pregnancy
LBD: ligand binding domain
MDR3/Mdr2: multidrug resistance protein 3/2
PM5S: epiallopregnanolone sulfate
