# Supplementary material for: Intrahepatic Cholestasis of Pregnancy Levels of Sulfated Progesterone Metabolites Inhibit Farnesoid X Receptor Resulting in a Cholestatic Phenotype
Source: Hepatology. 2013 Jan 8;57(2):716–26. doi: 10.1002/hep.26055 (PMC3592994; doi:10.1002/hep.26055)
Supplement: Supplementary file 1 [file hep0057-0716-SD1.pdf]

Manuscript Number HEP-12-0720

## Supporting Information

### Supplementary Materials

***Reagents***. All bile acids and steroid compounds were purchased from Steraloids; $^3$H-taurocholic acid ($^3$HTC; specific activity 5Ci/mmol) and UltimaGold scintillation cocktail were purchased from PerkinElmer. All chemicals were purchased from Sigma-Aldrich, unless otherwise stated.

***Steroid Assays in Human and Mouse Serum.*** Serum PM5S was analyzed by ultra-performance liquid chromatography-tandem mass spectrometry (UPLC/MSMS) using a modified and extended methodology initially for bile acid analysis in human serum.[19]

***Cell Culture.*** Huh7 cells were obtained from Dr. Clare Selden (UCL Centre for Hepatology, University College Medical School, London, UK) and routinely subcultured in Dulbecco's modified Eagle's medium (DMEM) supplemented with 10% fetal calf serum, antibiotic/antimycotic and L-glutamine (Invitrogen). For experiments involving transfections/treatments, Huh7 cells were subcultured in phenol-red free DMEM supplemented with 2.5% dextran-coated charcoal (DCC) treated fetal calf serum (Hyclone) for 24 hours.

For the preparation of primary human hepatocytes (PHH), human liver tissue was taken (with informed consent and local research ethics approval (RFH38–2000)) at tumor-free resection margins following surgical intervention for secondary liver tumors. PHH were isolated by collagenase perfusion.[19] Isolated hepatocytes were plated into 24-well type I collagen-coated plates (BD Biosciences) using hepatocyte basal media with supplements (Lonza).

***Huh7 $^3$H-taurocholic acid ($^3$HTC) Efflux***. Huh7 cells that were seeded into 24-well plates and incubated overnight with GW4064 ±PM5S were washed with 2.5% DCC phenol red free DMEM for 1.5 hours, the media being replaced every 15 minutes. Cells were then incubated at 37$^o$C with 250µl of influx media consisting of 0.5µM $^3$HTC and 0.5µM sodium taurocholic acid in phenol red free DMEM supplemented with 2.5% DCC for 30 minutes. At this point, cells were either progressed to the efflux component of the experiment or assessed for intracellular $^3$HTC levels.

For the assessment of intracellular $^3$HTC levels, cells were washed three times with 500µl of ice-cold 1mM sodium taurocholate and lysed with 250µl of RIPA buffer (Sigma-Aldrich), 175µl of which were mixed with 2ml of scintillation cocktail for radioactivity counting and the remainder used to assay protein quantity (Pierce BCA) for normalization.

To assess Huh7 cell efflux activity, wells were washed three times with ice cold PBS. Cells were then incubated with 125µl of phenol red free DMEM supplemented with 2.5% DCC for five minutes. 120µl of efflux media was then removed from the well and mixed with 2 ml of scintillation cocktail for radioactivity counting. Following the efflux period, cells were washed twice with cold D-PBS and lysed in 200µl of RIPA buffer and protein quantity assayed.

***RNA Isolation and Quantitative PCR***. Total RNA was isolated (Qiagen RNeasy kit) from Huh7 cells and PHH. 0.5µg of RNA was reverse-transcribed using the Advantage RT-for-PCR kit (Takara Biosciences). Expression levels of genes of interest were assayed by quantitative PCR using SYBR Green Jumpstart Readymix (Sigma) and calculated using the $\Delta\Delta$Ct method. Sequences of the primers used for qPCR assays are available upon request.

***Preparation of GST-FXR (LBD) Fusion Protein.*** Escherichia coli strain BL21 (Stratagene) harboring pGST-hFXR-ligand binding domain (LBD) was cultured in LB medium to a density of $A_{600}$ 0.7–1.0 and induced for overexpression by the addition of isopropyl-β-D-thiogalactopyranoside to a final concentration of 0.2mM. The isopropyl-β-D-thiogalactopyranoside-induced cultures were grown at room temperature for an additional 2–5 h. The cells were harvested by centrifugation for 10 min at 5000 × g. The cell pellet was used for GST fusion protein purification according to the recommended procedure from GE Healthcare using GSTrap HP and HiTrap desalting columns.

***Plasmids.*** pcDNA-RXR, pcDNA-FXRα2, pGL3-IBAP-Luc, pGL3-IBAP-Luc and pCMV-Renilla have been described elsewhere.[4] GST-FXR-LBD was constructed by cloning the FXRα2 LBD (residue 244-Glu to residue 472-Gln) into pGEX-6P2 BamH1/Not1 restriction sites. Sequences of the primers used for cloning are available upon request.

***Western Blot Analysis***. Huh7 cells seeded into 6-well plates were lysed in 150µl of lysis solution (1mM EDTA pH8.0, 1% SDS (W/V)) and sonicated. 30µg of protein was run on a 4-12% bis-tris acrylamide gradient gel (Invitrogen) and transferred onto a nitrocellulose membrane. Membranes were blocked with 5% milk in 0.1% TBS-Tween-20 and BSEP probed with a 1/100 dilution of rabbit polyclonal anti-ABCB11 antibody (Abcam ab71793) or with a 1/20000 dilution of mouse monoclonal anti-GAPDH (Millipore MAB374). Membranes were then incubated with HRP-conjugated secondary antibodies (Santa Cruz) and developed with SuperSignal West PICO Chemiluminescent substrate (ThermoFisher).

***Transactivation Assays.*** Huh7 cells seeded into 96-well plates were transfected with 10.4ng pcDNA-RXR, 10.4ng pcDNA-FXRα2 / pcDNA3.1 or pSG5 together with 10.4ng and 40ng pGL3-IBAP-Luc / pGL3-BSEP-Luc and pCMV-Renilla using Fugene 6 transfection reagent (Roche) at a 3:1 ratio. 24 hours later, cells were washed and treated with 0-200µM compound in the presence or absence of 100µM CDCA. After 24 hours, the luciferase activity was determined using Steadylite plus (PerkinElmer) and Renilla luciferase activity used as internal control was determined by the addition of EDTA (8mM final concentration) and Coelenterazine substrate (Calbiochem), and measured with a Victor2 luminometer (PerkinElmer) to correct for the transfection efficiency. Transfection experiments were performed at least three times, and the results are shown as mean values of quadruplicates and standard deviations.

***FXR Co-activator Recruitment Assays.*** A homogeneous time-resolved fluorescence (HTRF)-based FXR and co-activator SRC-1 interaction assay was used to examine the interaction of FXR with various ligands according to methods previously described for other nuclear receptors[37] with minor modifications. Briefly, 16µl of reaction mixture (50mM HEPES, 125mM KF, 0.125% (w/v) CHAPS, 0.05% dry milk, 4nM GST-FXR LBD, 1nM anti-GST-(Eu)K (Cisbio), 10nM biotin-SRC-1 (residues 676–700) (AnaSpec catalogue 62152), 20nM SA/XL665 (Cisbio)) were added to each well, followed by the addition of 4µl of $Me_2SO$ (6% v/v in 20µl reaction) or ligands (in $Me_2SO$) into appropriate wells in the presence or absence of 9/100µM sodium chenodeoxycholate in 384-well plates that were incubated at room temperature on an orbital shaker for 1 hour. HTRF measurements were read on a PHERAstar Plus (BMG LABTECH). Data were expressed as the ratio of the emission intensity at 665 nm to that at 620 nm multiplied by a factor of $10^4$. HTRF experiments were performed at least three times, and the results are shown as mean values of duplicates and standard deviations.

***Small Interfering RNA Transfection.*** 100nM of Dharmacon ON-TARGETplus SMARTpool against FXR (catalogue L-003414) or siGENOME non-targeting siRNA 4 (catalogue D-001210-04) at a final concentration of 100nM were transfected into PHH using Dharmafect 1 reagent following the manufacturer's guidelines for 6 hours. PHH transfection media was replaced with hepatocyte basal media and cells allowed to recover overnight. Cells were treated with compound for 24 hours before being harvested.

***Graphing and Statistics Composition.*** OriginPro 8 software (OriginLab Corp.) was used to generate graphical representations and to calculate $EC_{50}$ and $IC_{50}$ values (dose-response fit), and statistical significance of the differences between groups was determined by Student's paired/unpaired t-test or one-way ANOVA. Results are expressed as mean ± S.D., unless otherwise stated.

## Supporting Figures

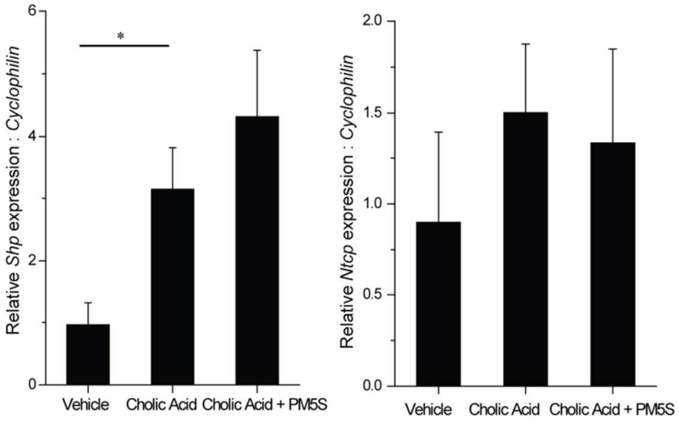


**Supporting Figure 1.** PM5S does not perturb cholic acid-mediated gene expression profiles for *Shp* and *Ntcp*. qPCR analysis of *Shp* and *Ntcp* gene expression in mice gavaged vehicle, cholic acid or cholic acid and PM5S. *, $P < 0.05$ for treatment group versus vehicle control. Statistical significance was determined by one-way ANOVA. Values represent mean ± S.E.M. of n=6.

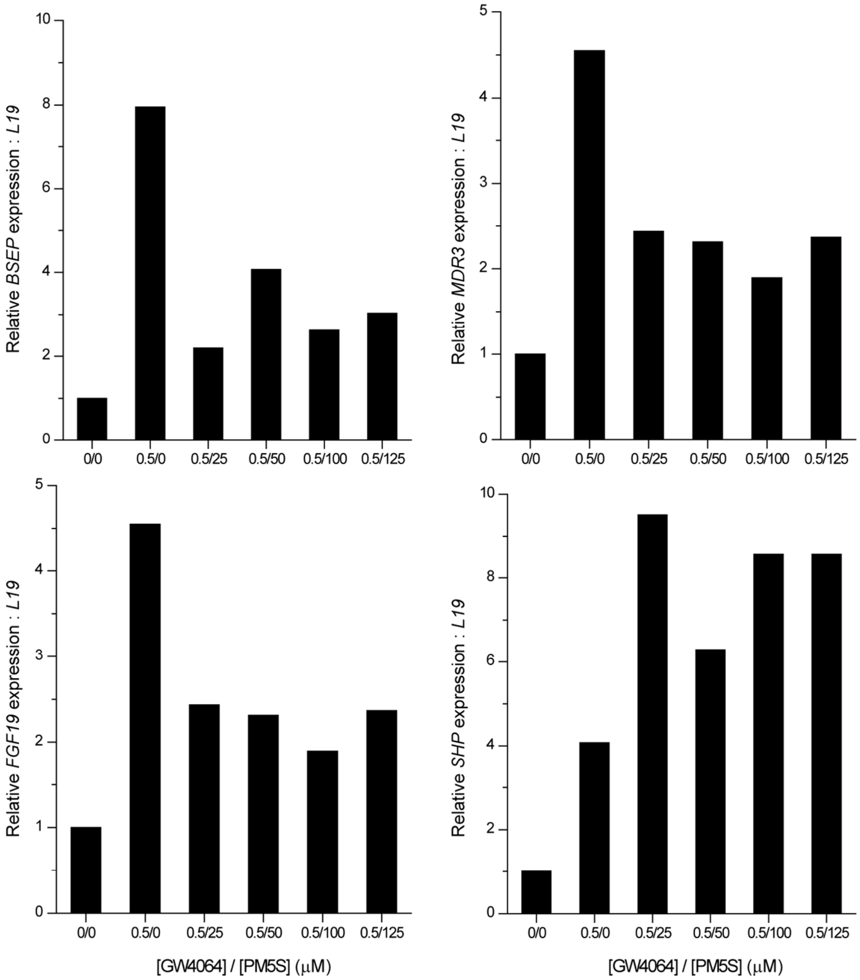


**Supporting Figure 2.** PM5S inhibits GW4064-mediated expression of FXR target genes. (*A*) Representative primary human hepatocyte culture that was treated with vehicle or 0.5μM GW4064 ±0-125μM PM5S for 24 hours after which cells were analyzed with qPCR for relative *BSEP, MDR3, FGF19 and SHP* gene expression.

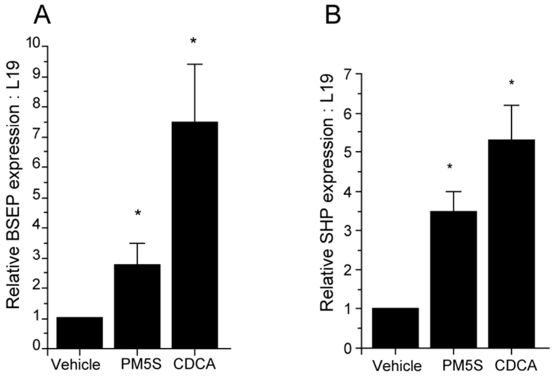


**Supporting Figure 3.** PM5S treatment results in mild *BSEP* and *SHP* induction. (*A*) Primary human hepatocytes were treated with 50μM PM5S or 50μM CDCA for 24 hours and analyzed with qPCR for relative *BSEP* gene expression. *, P < 0.05 for treatment group versus vehicle control. (*B*) PM5S exhibits mild *SHP* agonist activity. Experiments were performed as in *D*. *, P < 0.05 for treatment group versus vehicle control. Statistical significance was determined by one-way ANOVA. Values represent mean ± S.E.M. of n=3.

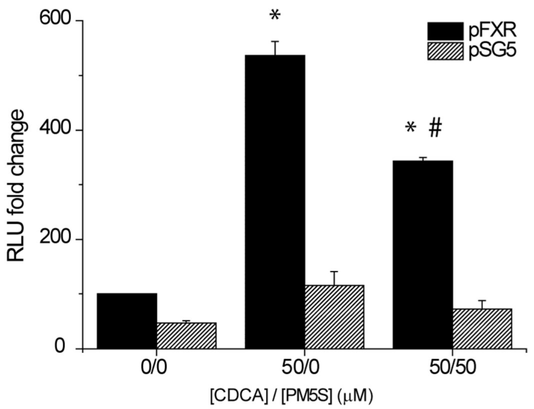


**Supporting Figure 4.** PM5S reduces ligand-mediated FXR transactivity at the BSEP promoter. (A) Huh7 cells were transfected with the human expression constructs RXR, FXRα2 / empty vector, BSEP promoter-luciferase reporter and renilla construct for 24 hours. Transfected cells were treated with vehicle (0/0 group) or 50μM CDCA ±50μM PM5S. RLU, relative light units. *, P < 0.05 for vehicle group versus 50μM CDCA / co-treatment group. #, P < 0.05 for 50μM CDCA versus co-treatment group. Statistical significance was determined by one-way ANOVA. n=3 ±S.E.M.

| **Trivial name** | **Abbreviation** | **Name** | **Compound structure** |
| --- | --- | --- | --- |
| Allopregnanolone | PM4 | 5α-pregnan-3α-ol-20-one | 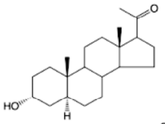 |
| Epiallopregnanolone | PM5 | 5α-pregnan-3β-ol-20-one | 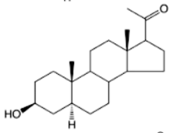 |
| Allopregnanolone acetate | PM4a | 5α-pregnan-3α-ol-20-one acetate | 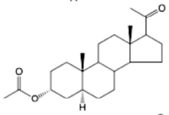 |
| Epiallopregnanolone acetate | PM5a | 5α-pregnan-3β-ol-20-one acetate | 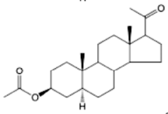 |
| Allopregnanolone sulphate | PM4S | 5α-pregnan-3α-ol-20-one sulphate | 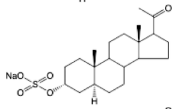 |
| Epiallopregnanolone sulphate | PM5S | 5α-pregnan-3β-ol-20-one sulphate | 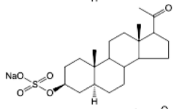 |
| Pregnanolone sulphate | PS | 5β-pregnan-3α-ol-20-one sulphate | 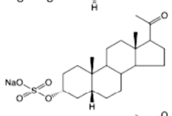 |
| Epipregnanolone sulphate | EPS | 5β-pregnan-3β-ol-20-one sulphate | 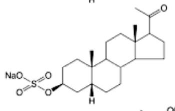 |
| Epiallo-pregnanediol 3-sulphate | EPAS | 5α-pregnan-3β, 20β-diol-3-sulphate | 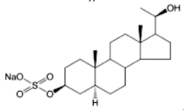 |
| Pregnanediol glucuronide | PG | 5β-pregnan-3α, 20α-diol 3-glucosiduronate | 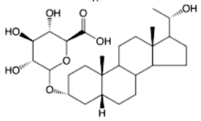 |
| Chenodeoxycholic acid | CDCA | 5β-cholanic acid-3α, 7α-diol | 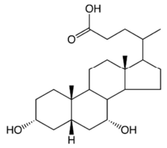 |

**Supporting Figure 5.** List of progesterone based compounds used in Figure 5*A* ligand screen.

| Sample | Gestation (weeks) | Itch | UDCA | Sample ALT* (IU/L) | Sample Bilirubin* (μM) | Sample Total BA* (μM) | Maximal ALT Level in Pregnancy (IU/L) | Maximal BA Level in Pregnancy (μM) |
|---|---|---|---|---|---|---|---|---|
| C1 | 35.7 | NA | NA | NA | NA | 2 | NA | NA |
| C3 | 35 | NA | NA | NA | NA | 1 | NA | NA |
| C4 | 33.6 | NA | NA | NA | NA | 1 | NA | NA |
| C6 | 34.2 | NA | NA | NA | NA | 2 | NA | NA |
| C7 | 38.9 | NA | NA | NA | NA | 2 | NA | NA |
| C8 | 41.0 | NA | NA | NA | NA | 3 | NA | NA |
| C9 | 36.3 | NA | NA | NA | NA | 1 | NA | NA |
| C10 | 38.9 | NA | NA | NA | NA | 3 | NA | NA |
| C11 | 39.3 | NA | NA | NA | NA | 6 | NA | NA |
| C12 | 37.7 | NA | NA | NA | NA | 2 | NA | NA |
| | | | | | | | | |
| ICP1 | 33.4 | Y | Y | ND | ND | 129 | 33 | 129 |
| ICP2 | 35.5 | Y | Y | 128 | 8 | 19 | 269 | 21 |
| ICP3 | 34.1 | Y | Y | 419 | 12 | 8 | 419 | 92 |
| ICP4 | 35.3 | Y | N | 164 | 9 | 57 | 554 | 64 |
| ICP5 | 32.6 | Y | Y | 71 | 27 | 42 | 71 | 157 |
| ICP6 | 35.4 | Y | Y | 10 | 6 | 4 | 15 | 21 |
| ICP7 | 34.7 | Y | N | ND | 11 | 14 | 65 | 130 |
| ICP8 | 37.4 | Y | N | 36 | 4 | 23 | 43 | 34 |
| ICP9 | 36.1 | Y | N | 17 | 5 | 6 | 17 | 25 |
| ICP10 | 36.6 | Y | N | 23 | 9 | 2 | 28 | 22 |
| ICP11 | 38.7 | Y | N | 16 | 9 | 6 | 45 | 39 |
| ICP12 | 35.6 | Y | Y | 149 | 18 | 57 | 285 | 57 |
| ICP13 | 35.4 | Y | N | 63 | 6 | 24 | 87 | 35 |
| ICP14 | 34.1 | Y | N | 99 | 7 | 20 | 496 | >150 |
| ICP15 | 35.5 | Y | Y | 6 | 6 | 16 | 11 | 40 |

Supporting Table 1. The clinical and biochemical features of the intrahepatic cholestasis of pregnancy (ICP) cases from whom serum was obtained for the determination of PM5S levels. C represents control and ICP represents intrahepatic cholestasis of pregnancy. *, these values represent the level of ALT, bilirubin and total bile acid levels in the sample that was used for the quantification of PM5S using LCMS. NA – Not Applicable; Y – Yes; N – No; ND – Not Determined; BA – Bile Acids; ALT – alanine transaminase.

Supporting Table 2. Target and L19 Ct qPCR values taken from Huh7 and primary human hepatocytes treated with 0.5μM GW4064

| | Huh7 | | PHH | |
|---|---|---|---|---|
| Target | Target Ct | L19 Ct | Target Ct | L19 Ct |
| *BSEP* | 21.2 | 17.7 | 22.6 | 20.8 |
| *FGF19* | 20.1 | 17.7 | 21.9 | 20.8 |
| *SHP* | 20.6 | 17.7 | 21.4 | 20.8 |
| *MDR3* | 21.1 | 17.7 | 19.8 | 20.8 |
| *FXR* | 22.1 | 17.7 | 23.6 | 20.8 |
